# Special Issue: Innovations in Semiconducting Block Copolymers

**DOI:** 10.3390/ma15186390

**Published:** 2022-09-14

**Authors:** Francesca Villafiorita-Monteleone, Stefania Zappia

**Affiliations:** Istituto di Scienze e Tecnologie Chimiche “Giulio Natta” (SCITEC), Consiglio Nazionale delle Ricerche (CNR), Sede Via A. Corti 12, 20133 Milano, Italy

The current Special Issue entitled “Innovations in Semiconducting Block Copolymers” aims to discuss cutting-edge research regarding the synthesis, characterization and application of semiconducting block copolymers, with a special focus on the realization of novel and innovative nanostructured materials for the production of advanced devices suitable in different fields, ranging from sensors applications to optic photovoltaics.

Block copolymers have long been exploited to obtain thin-film architectures with controlled morphology, thanks to their capacity to self-assemble [1]. In fact, a variety of nanoscale morphologies have been reported, such as lamellar, spherical, cylindrical, vesicular and microporous structures [2]. Moreover, it has been demonstrated that semiconducting block copolymers can be used as active layers in OPV devices and solar cells, thanks to the possibility to obtain a continuous phase of donor and acceptor components in the desired nanometer domain scale [3]. In this context, the self-assembly capability of peculiar semiconducting block copolymers has been demonstrated in aqueous media, leading to the preparation of water-processable nanoparticles (NPs) composed of a blend containing a semiconducting polymer and a fullerene derivative. This original approach recently emerged as a smart strategy to control the nanoscale morphology and reduce the use of harmful solvents [4,5,6].

The research interest of this Special Issue thus includes, but is not limited to, the following: production and characterization of nanostructured and semiconducting active materials; study of the hierarchical organization of block copolymer nanostructures; device production and characterization. Moreover, the study of the structure–morphology relationship of semiconducting block copolymers, neat or in blend with other materials to achieve complex composites with innovative properties, is a central topic of this Special Issue.
